# Supplementary material for: Development of nitrogen efficiency screening system in alfalfa (Medicago sativa L.) and analysis of alfalfa nitrogen efficiency types
Source: PeerJ. 2022 May 6;10:e13343. doi: 10.7717/peerj.13343 (PMC9083526; doi:10.7717/peerj.13343)
Supplement: Supplemental Information 2 [file peerj-10-13343-s002.docx]

**Table S1** Alfalfa cultivars and their source, distribution and origin

| **Number** | **Cultivars** | **Source** | **Distribution area** | **Origin** |
| --- | --- | --- | --- | --- |
| 1 | Gannong NO.3 | College of Prataculture Science, GAU | Northern area | China |
| 2 | Gannong NO.4 | Gansu Agricultural University College of Prataculture | Northern area | China |
| 3 | Gannong NO.5 | Gansu Academy of Agricultural Sciences | Northern area | China |
| 4 | Gannong NO.7 | Gansu Agricultural University College of Prataculture | Northern area | China |
| 5 | Gannong NO.8 | Gansu Agricultural University College of Prataculture | Northern area | China |
| 6 | Gannong NO.9 | Gansu Agricultural University College of Prataculture | Northern area | China |
| 7 | Longdong | Gansu Agricultural University College of Prataculture | Northern area | China |
| 8 | Xinjiangdaye | Gansu Agricultural University College of Prataculture | Northern area | China |
| 9 | Xinmu NO.1 | College of Prataculture and Environmental Science, Xinjiang Agricultural University | Northern area | China |
| 10 | Longmu 806 | College of Prataculture and Environmental Science, Xinjiang Agricultural University | Northern area | China |
| 11 | Longmu 801 | Heilongjiang Institute of Animal Husbandry | Northern area | China |
| 12 | Gongnong NO.1 | Animal Husbandry Branch, Jilin Academy of Agricultural Sciences | Northern area | China |
| 13 | Gongnong NO.3 | Animal Husbandry Branch, Jilin Academy of Agricultural Sciences | Northern area | China |
| 14 | Zhaodong | College of Prataculture and Environmental Science, Xinjiang Agricultural University | Northern area | China |
| 15 | LW6010 | Purple pasture Co., Ltd. | Southern area | America |
| 16 | Reindeer | Beijing Kravo Grass Technology Development Center | Most parts of the north area | Canada |
| 17 | Crown | Beijing Kravo Grass Technology Development Center | Most parts of the north area | America |
| 18 | Goldqueen | College of Prataculture and Environmental Science, Xinjiang Agricultural University | Northern area | America |
| 19 | Giant 551 | Beijing Kravo Grass Technology Development Center | Temperate area | America |
| 20 | Giant 601 | Beijing Kravo Grass Technology Development Center | Temperate and warm temperate area | America |
| 21 | Giant 6 | Beijing Kravo Grass Technology Development Center | Northern temperate area | America |
| 22 | Giant 2 | Beijing Kravo Grass Technology Development Center | Northern cold area | America |
| 23 | Sadie 7 | Purple pasture Co., Ltd | Southern area | Australia |
| 24 | Sadie 10 | Purple pasture Co., Ltd | Southern area | Australia |
| 25 | Tourists | Purple pasture Co., Ltd | Southern area | Australia |
| 26 | Elite | Purple pasture Co., Ltd | Southern area | America |
| 27 | Weston | Purple pasture Co., Ltd | Subtropical area | America |
| 28 | Algonquin | College of Prataculture and Environmental Science, Xinjiang Agricultural University | Northern area | Canada |
