# Supplementary material for: Development of nitrogen efficiency screening system in alfalfa (Medicago sativa L.) and analysis of alfalfa nitrogen efficiency types
Source: PeerJ. 2022 May 6;10:e13343. doi: 10.7717/peerj.13343 (PMC9083526; doi:10.7717/peerj.13343)
Supplement: Supplemental Information 4 — Note: SDW, RDW, PDW, SH, RL, RV, SNC, RNC, PNC, SNA, RNA and PNA respective shoot dry weight, root dry weight, plant dry weight, plant height, total root length, total root volume, shoot nitrogen content, root nitrogen content, plant nitrogen content, shoot nitrogen accumulation, root nitrogen accumulation and plant nitrogen accumulation. [file peerj-10-13343-s004.docx]

**Table S3** Changes of 28 alfalfa cultivars under medium nitrogen level (N210) in seedling stage

| **NO.** | **Cultivars** | **SDW**  **(g•plant ^-1^)** | **RDW**  **(g•plant ^-1^)** | **PDW**  **(g•plant ^-1^)** | **SH**  **(cm)** | **RL**  **(cm•plant ^-1^)** | **RV**  **(cm^3^)** | **SNC**  **(%)** | **RNC**  **(%)** | **PNC**  **(%)** | **SNA**  **(mg•plant ^-1^)** | **RNA**  **(mg•plant ^-1^)** | **PNA**  **(mg•plant ^-1^)** |
| --- | --- | --- | --- | --- | --- | --- | --- | --- | --- | --- | --- | --- | --- |
| 1 | Gannong NO.3 | 2.23±0.22^e^ | 0.24±0.01^c^ | 2.47±0.21d^efg^ | 15.17±0.21^g^ | 90.13±18.66^abc^ | 0.08±0.02^abcd^ | 2.14±0.02^o^ | 2.78±0.05^ij^ | 2.21±0.02^mm^ | 4.78±0.48^ghi^ | 0.67±0.03^fg^ | 5.44±0.47^ijk^ |
| 2 | Gannong NO.4 | 2.19±0.09^e^ | 0.14±0.02i^jk^ | 2.33±0.09^g^ | 15.90±0.15^ef^ | 71.79±9.69^bcdef^ | 0.04±0.01^hi^ | 2.96±0.15^gh^ | 3.49±0.01^c^ | 2.99±0.14^gh^ | 6.49±0.26^de^ | 0.48±0.05^jklm^ | 6.97±0.27^fg^ |
| 3 | Gannong NO.5 | 2.25±0.07^e^ | 0.20±0.00^def^ | 2.44±0.07^efg^ | 17.68±0.15^b^ | 75.13±5.71^abcde^ | 0.08±0.01^ab^ | 3.36±0.04^bc^ | 3.1±0.07^gh^ | 3.34±0.03^cd^ | 7.54±0.23^c^ | 0.61±0.01^g^ | 8.15±0.23^c^ |
| 4 | Gannong NO.7 | 1.34±0.03^k^ | 0.19±0.00^efg^ | 1.53±0.04^n^ | 14.82±0.15^h^ | 78.25±11.62^abcde^ | 0.06±0.01^bcdefgh^ | 3.25±0.04^cd^ | 3.24±0.02^e^ | 3.25±0.03^de^ | 4.36±0.11^i^ | 0.61±0.01^g^ | 4.96±0.12^k^ |
| 5 | Gannong NO.8 | 1.15±0.09^l^ | 0.2±0.01^def^ | 1.35±0.09^p^ | 14.80±0.15^h^ | 80.14±8.15^abcd^ | 0.05±0.01^efghi^ | 2.28±0.08^mn^ | 3.45±0.02^c^ | 2.45±0.08^l^ | 2.62±0.21^l^ | 0.68±0.03^fg^ | 3.30±0.22^m^ |
| 6 | Gannong NO.9 | 1.43±0.03^k^ | 0.19±0.00^efg^ | 1.62±0.03^mn^ | 13.72±0.15^g^ | 98.97±8.79^a^ | 0.06±0.01^bcdefgh^ | 3.19±0.02^de^ | 3.3±0.01^d^ | 3.20±0.02^e^ | 4.57±0.11^hi^ | 0.62±0.01^g^ | 5.19±0.10^jk^ |
| 7 | Longdong | 1.72±0.04^hij^ | 0.15±0.02^hi^ | 1.88±0.03^ijk^ | 13.38±0.15^k^ | 30.11±0.96^g^ | 0.02±0.00^i^ | 2.12±0.02^o^ | 2.64±0.02^k^ | 2.17±0.02^n^ | 3.66±0.08^j^ | 0.41±0.05^lmno^ | 4.07±0.07^l^ |
| 8 | Xinjiangdaye | 2.42±0.03^bcd^ | 0.17±0.02^gh^ | 2.59±0.03^cde^ | 15.65±0.21^f^ | 79.93±21.04^abcd^ | 0.09±0.04^a^ | 2.80±0.05^jk^ | 3.15±0.01^fg^ | 2.82±0.04^jk^ | 6.78±0.08^d^ | 0.53±0.06^ij^ | 7.31±0.07^ef^ |
| 9 | Xinmu NO. 1 | 2.53±0.09^bcd^ | 0.22±0.01^def^ | 2.75±0.1^b^ | 17.35±0.21^c^ | 61.64±7.03^def^ | 0.05±0.00^defghi^ | 2.48±0.02^l^ | 3.44±0.06^c^ | 2.55±0.02^l^ | 6.27±0.21^ef^ | 0.75±0.04^def^ | 7.02±0.25^fg^ |
| 10 | Longmu 806 | 2.25±0.09^e^ | 0.12±0.00^jkl^ | 2.37±0.09^fg^ | 15.98±0.21^e^ | 54.67±14.04^ef^ | 0.06±0.02^bcdefgh^ | 3.36±0.03^bc^ | 3.59±0.00^b^ | 3.37±0.03^bc^ | 7.56±0.30^c^ | 0.44±0.01^klmn^ | 8.00±0.29^cd^ |
| 11 | Longmu 801 | 1.84±0.09^fgh^ | 0.12±0.01^kl^ | 1.96±0.09^ij^ | 16.35±0.21^d^ | 64.07±14.23^def^ | 0.06±0.01^abcdefgh^ | 2.84±0.07^hijk^ | 3.33±0.03^d^ | 2.87±0.07^ij^ | 5.23±0.25^g^ | 0.38±0.04^no^ | 5.61±0.27^ij^ |
| 12 | Gongnong NO.1 | 1.66±0.03^ij^ | 0.14±0.01^ijk^ | 1.8±0.04^jkl^ | 12.58±0.15m | 81.77±22.32^abcd^ | 0.07±0.03^abcde^ | 2.87±0.04^hij^ | 3.69±0.01^a^ | 2.93±0.04^hij^ | 4.77±0.10^ghi^ | 0.50±0.03^ijk^ | 5.27±0.12^ijk^ |
| 13 | Gongnong NO.3 | 0.97±0.09^m^ | 0.13±0.00^jkl^ | 1.09±0.09^p^ | 14.72±0.15^h^ | 66.61±18.22^cdef^ | 0.05±0.02^defghi^ | 2.82±0.03^ijk^ | 2.82±0.05^i^ | 2.82±0.02^jk^ | 2.73±0.24^l^ | 0.35±0.01^o^ | 3.08±0.24^m^ |
| 14 | Zhaodong | 2.33±0.04^cde^ | 0.24±0.00^c^ | 2.57±0.04^cde^ | 15.00±0.15^gh^ | 77.53±7.73^abcde^ | 0.05±0.01^cdefgh^ | 2.96±0.11^gh^ | 3.30±0.01^d^ | 2.99±0.10^gh^ | 6.90±0.11^d^ | 0.80±0.01^de^ | 7.70±0.11^de^ |
| 15 | LW6010 | 2.84±0.07^a^ | 0.46±0.02^a^ | 3.30±0.05^a^ | 18.10±0.21^a^ | 70.37±6.99^bcdef^ | 0.05±0.01^efghi^ | 3.44±0.03^b^ | 3.65±0.04^a^ | 3.47±0.03^ab^ | 9.77±0.23^a^ | 1.69±0.07^a^ | 11.46±0.17^a^ |
| 16 | Reindeer | 1.66±0.09^ij^ | 0.11±0.00^kl^ | 1.78±0.09^kl^ | 16.48±0.21^d^ | 61.87±11.34^def^ | 0.06±0.02^bcdefgh^ | 3.11±0.05^ef^ | 3.55±0.04^b^ | 3.14±0.05^ef^ | 5.16±0.27^g^ | 0.41±0.01^mno^ | 5.57±0.27^ij^ |
| 17 | Crown | 2.46±0.16^bc^ | 0.25±0.00^c^ | 2.72±0.16^bc^ | 15.90±0.15^ef^ | 95.01±7.23^ab^ | 0.06±0.00^abcdefgh^ | 3.24±0.01^d^ | 3.23±0.04^e^ | 3.24±0.01^de^ | 7.98±0.52^bc^ | 0.82±0.01^d^ | 8.80±0.53^b^ |
| 18 | Goldqueen | 1.97±0.06^f^ | 0.15±0.01^hij^ | 2.12±0.07^h^ | 16.48±0.21^d^ | 61.68±14.92^def^ | 0.06±0.02^bcdefgh^ | 3.03±0.05^fg^ | 3.33±0.03^d^ | 3.05±0.05^fg^ | 5.99±0.19^f^ | 0.49±0.05^ijkl^ | 6.48±0.22^h^ |
| 19 | Giant 551 | 1.91±0.04^fg^ | 0.11±0.00^l^ | 2.02±0.04^hi^ | 14.70±0.15^h^ | 54.71±7.21^ef^ | 0.04±0.01^fghi^ | 2.72±0.04^k^ | 3.10±0.01^gh^ | 2.74±0.04^k^ | 5.21±0.10^g^ | 0.33±0.01^o^ | 5.55±0.10^ij^ |
| 20 | Giant 601 | 2.31±0.17^cde^ | 0.19±0.00^fg^ | 2.50±0.17^def^ | 17.17±0.21^c^ | 78.64±16.63^abcde^ | 0.07±0.01^abcdef^ | 3.57±0.03^a^ | 3.69±0.01^a^ | 3.57±0.03^a^ | 8.25±0.61^b^ | 0.68±0.01^fg^ | 8.93±0.62^b^ |
| 21 | Giant 6 | 1.62±0.09^ij^ | 0.21±0.01^def^ | 1.83±0.09^jkl^ | 15.67±0.15^ef^ | 62.05±3.52^def^ | 0.04±0.01^ghi^ | 2.93±0.04^ghi^ | 2.74±0.01^j^ | 2.91±0.04^hij^ | 4.75±0.27^ghi^ | 0.57±0.02^hi^ | 5.32±0.26^ijk^ |
| 22 | Giant 2 | 2.26±0.09^de^ | 0.35±0.05^b^ | 2.61±0.08^bcd^ | 14.35±0.21^i^ | 69.97±0.63^cdef^ | 0.07±0.02^abcdefg^ | 3.37±0.02^b^ | 3.56±0.04^b^ | 3.39±0.02^bc^ | 7.62±0.29^c^ | 1.24±0.16^b^ | 8.86±0.26^b^ |
| 23 | Sadie 7 | 0.57±0.03^n^ | 0.21±0.01^de^ | 0.78±0.04^q^ | 12.3±0.15^m^ | 75.68±14.86^abcde^ | 0.07±0.01^abcdefgh^ | 2.45±0.02^l^ | 3.47±0.01^c^ | 2.72±0.03^k^ | 1.39±0.08^m^ | 0.73±0.02^ef^ | 2.12±0.10^n^ |
| 24 | Sadie 10 | 1.95±0.09^f^ | 0.16±0.00^gh^ | 2.11±0.09^h^ | 14.27±0.15^i^ | 62.53±7.97^def^ | 0.04±0.01^ghi^ | 3.16±0.03^de^ | 3.05±0.03^h^ | 3.15±0.03^ef^ | 6.17±0.27^ef^ | 0.50±0.01^ijk^ | 6.67±0.27^gh^ |
| 25 | Tourists | 0.64±0.04^n^ | 0.17±0.01^gh^ | 0.81±0.04^q^ | 15.98±0.21^e^ | 50.14±13.40^fg^ | 0.07±0.01^abcdef^ | 2.19±0.02^no^ | 2.65±0.02^k^ | 2.28±0.01^mm^ | 1.40±0.08^m^ | 0.45±0.02^jklmn^ | 1.85±0.10^n^ |
| 26 | Elite | 1.59±0.03^j^ | 0.13±0.00^jkl^ | 1.71±0.03l^m^ | 13.03±0.15^l^ | 75.58±2.32^abcde^ | 0.07±0.01^abcde^ | 3.14±0.17^def^ | 3.23±0.04^e^ | 3.15±0.16^ef^ | 4.99±0.11^gh^ | 0.40±0.01^mno^ | 5.39±0.10^ijk^ |
| 27 | Weston | 1.35±0.09^k^ | 0.33±0.02^b^ | 1.68±0.1l^m^ | 13.03±0.21^l^ | 71.56±17.20^bcdef^ | 0.08±0.02^abc^ | 2.38±0.01^lm^ | 3.18±0.02^ef^ | 2.54±0.01^l^ | 3.22±0.21^k^ | 1.06±0.06^c^ | 4.28±0.27^l^ |
| 28 | Algonquin | 1.78±0.09^ghi^ | 0.15±0.01^hi^ | 1.93±0.09^ijk^ | 16.47±0.15^d^ | 77.90±16.46^abcde^ | 0.05±0.02^cdefgh^ | 2.94±0.17^ghi^ | 3.15±0.05^fg^ | 2.95±0.16^ghi^ | 5.22±0.25^g^ | 0.47±0.03^jklm^ | 5.69±0.28^i^ |

Note: SDW, RDW, PDW, SH, RL, RV, SNC, RNC, PNC, SNA, RNA and PNA respective shoot dry weight, root dry weight, plant dry weight, plant height, total root length, total root volume, shoot nitrogen content, root nitrogen content, plant nitrogen content, shoot nitrogen accumulation, root nitrogen accumulation and plant nitrogen accumulation.
